# Predicting RNA secondary structure by the comparative approach: how to select the homologous sequences

**DOI:** 10.1186/1471-2105-8-464

**Published:** 2007-11-28

**Authors:** Stéfan Engelen, Fariza Tahi

**Affiliations:** 1 Laboratoire IBISC – FRE CNRS 2873. CNRS, Université d'Evry Val-d'Essonne, Genopole. 523, place des Terrasses. 91000 Evry, France

## Abstract

**Background:**

The secondary structure of an RNA must be known before the relationship between its structure and function can be determined. One way to predict the secondary structure of an RNA is to identify covarying residues that maintain the pairings (Watson-Crick, Wobble and non-canonical pairings). This "comparative approach" consists of identifying mutations from homologous sequence alignments. The sequences must covary enough for compensatory mutations to be revealed, but comparison is difficult if they are too different. Thus the choice of homologous sequences is critical. While many possible combinations of homologous sequences may be used for prediction, only a few will give good structure predictions. This can be due to poor quality alignment in stems or to the variability of certain sequences. This problem of sequence selection is currently unsolved.

**Results:**

This paper describes an algorithm, *SSCA*, which measures the suitability of sequences for the comparative approach. It is based on evolutionary models with structure constraints, particularly those on sequence variations and stem alignment. We propose three models, based on different constraints on sequence alignments. We show the results of the *SSCA *algorithm for predicting the secondary structure of several RNAs. *SSCA *enabled us to choose sets of homologous sequences that gave better predictions than arbitrarily chosen sets of homologous sequences.

**Conclusion:**

*SSCA *is an algorithm for selecting combinations of RNA homologous sequences suitable for secondary structure predictions with the comparative approach.

## Background

Structural RNAs are important as regulators, catalysts, and structural components of cells. Their secondary structure must be known in order to understand the relationship between structure and function. The concept of secondary structure was introduced by Doty and Fresco [1]. The secondary structure is produced by the Watson-Crick pairings (AU and GC), Wobble pairing (GU), and non-canonical pairings [2]. The consecutive pairings form stems, and stems and loops make up the secondary structure; this, in turn forms the basis for tertiary structural elements like pseudoknots. The secondary structure of an RNA can be predicted from thermodynamic studies or by comparative studies. The thermodynamic approach is based on the idea that the actual structure is that with minimal free energy. This involves calculating the secondary structure of an RNA sequence that has the lowest free energy using a set of experimentally defined thermodynamic parameters [3,4]. The first efficient algorithm is based on dynamic programming [5]. An improvement of this algorithm was proposed by Zuker, who developed the Mfold program [6]. Mfold has a time complexity of O
 MathType@MTEF@5@5@+=feaafiart1ev1aaatCvAUfKttLearuWrP9MDH5MBPbIqV92AaeXatLxBI9gBaebbnrfifHhDYfgasaacPC6xNi=xH8viVGI8Gi=hEeeu0xXdbba9frFj0xb9qqpG0dXdb9aspeI8k8fiI+fsY=rqGqVepae9pg0db9vqaiVgFr0xfr=xfr=xc9adbaqaaeGacaGaaiaabeqaaeqabiWaaaGcbaWenfgDOvwBHrxAJfwnHbqeg0uy0HwzTfgDPnwy1aaceaqcfaOae8NdX=eaaa@37F1@(*n*^3^), where *n *is the length of the considered RNA sequence. A disadvantage of the thermodynamic approach is that the real structure is not necessarily the one with lowest free energy, but one that is close to this minimum. Mfold was therefore upgraded to find also suboptimal structures [7]. The thermodynamic approach is not so suitable for use with long sequences; the main problems are the influence of kinetics on folding and the lack of good thermodynamic parameters for junctions.

The comparative approach is used when several aligned homologous RNA sequences are available. The idea is to find pairs that covary to maintain Watson-Crick and Wobble complementarities (compensatory mutations) [8]. The comparative approach was initially used to predict manually the structure of long RNA sequences [8,9]. Later, some automatic procedures were proposed. The first algorithm was designed by Han and Kim [10]. However, all these early algorithms for implementing the comparative approach have high complexities (about O
 MathType@MTEF@5@5@+=feaafiart1ev1aaatCvAUfKttLearuWrP9MDH5MBPbIqV92AaeXatLxBI9gBaebbnrfifHhDYfgasaacPC6xNi=xH8viVGI8Gi=hEeeu0xXdbba9frFj0xb9qqpG0dXdb9aspeI8k8fiI+fsY=rqGqVepae9pg0db9vqaiVgFr0xfr=xfr=xc9adbaqaaeGacaGaaiaabeqaaeqabiWaaaGcbaWenfgDOvwBHrxAJfwnHbqeg0uy0HwzTfgDPnwy1aaceaqcfaOae8NdX=eaaa@37F1@(*n*^3^), where *n *is the length of the sequences). An algorithm implementing the comparative approach was proposed in [11] that has a complexity of *n*^2^*m*OMathType@MTEF@5@5@+=feaafiart1ev1aaatCvAUfKttLearuWrP9MDH5MBPbIqV92AaeXatLxBI9gBaebbnrfifHhDYfgasaacPC6xNi=xH8viVGI8Gi=hEeeu0xXdbba9frFj0xb9qqpG0dXdb9aspeI8k8fiI+fsY=rqGqVepae9pg0db9vqaiVgFr0xfr=xfr=xc9adbaqaaeGacaGaaiaabeqaaeqabiWaaaGcbaWenfgDOvwBHrxAJfwnHbqeg0uy0HwzTfgDPnwy1aaceaqcfaOae8NdX=eaaa@37F1@(*log*_4_*n*) in time with *n *the length of the sequence and *m *the number of homologous sequences used (*m *<< 10). The principle underlying this algorithm is to predict the secondary structure of a given sequence, the "target sequence", using a set of homologous sequences, the "test sequences". It uses the "divide and conquer" approach, searching for stems from the most significant to the least significant ones (introduced by Papanicolaou in [12]). The helices are selected according to criteria of length and number of compensatory mutations. Helices on the target sequence are first identified, and only those whose length is greater than or equal to *log*_4_*n *(where *n *is the length of the target sequence) are selected. The comparison step considers only the helices preserved in all the test sequences, with a minimum of compensatory mutations. This set of helices breaks down the target sequence into a set of sub-sequences. Other helices in each sub-sequence are then identified. This algorithm, *P-DCfold*, has been improved to find pseudoknots with the same complexity [13]. The thermodynamic approach is suitable when a few sequences (often one) are used, while the comparative approach is more appropriate for more sequences. Nevertheless, the two approaches can be combined [14,15]. The algorithm RNAalifold combines thermodynamic and covariation information in a modified energy model [16]. Another recent, novel algorithm, KNetFold [17], uses thermodynamic and mutual information [18]. This algorithm introduces a hierarchical network of k-nearest neighbor classifiers for predicting a consensus RNA secondary structure.

The drawback of the comparative approach is the need to use many homologous sequences. This approach becomes more useful as the number of available sequences increases. Nevertheless, it is difficult to select appropriated homologous sequences. Many of the algorithms for predicting the secondary structure of RNAs available today predict structure from an alignment and cannot accurately predict a structure from a very large alignment. A single misaligned sequence can destroy the prediction. The first part of this article shows that, while there are many possible combinations of homologous sequences that can be used, only a few correctly predict the structure. This can be due to the variability of homologous sequences and to poor quality alignment in stems (as shown in the second part of this paper). The problem of homologous sequence alignment can be overcome by predicting the structure and the alignment at the same time [19,20]. Another approach is to select the best set of homologous sequences for predicting the structure. But no publications up to now give any information on how this selection is done. We therefore assume that it is done manually. The third part of this paper describes an algorithm, *SSCA*, that makes this selection automatically. *SSCA *considers an alignment of a set of RNA homologous sequences, one of which is the "target sequence", and classifies the other sequences according to their suitability for predicting the structure of the target sequence. Finally, results of predictions made using the *SSCA *algorithm for sequence alignments of tmRNA, RNaseP and other RNAs are presented.

### Selecting homologous sequences

The secondary structure of a given sequence can be predicted from a set of aligned homologous sequences. The problem is to select those homologous sequences that are the most relevant for the comparison. This requires a set of well aligned homologous sequences, and at least two main problems can occur with the alignment of sequences under structure constraints:

• Regions at the ends of sequences are often quite variable, making it difficult or impossible to compare sequences in these regions. In contrast, the core of the structure is less variable (often because of catalytic activity), so that this region provides no useful information on compensatory mutations.

• Mutations are selected more rapidly in stem regions because of structure constraints. Thus, these regions seem to be more variable than the remainder of the structure (single strand regions) [21-23]. Consequently, alignments are often of poor quality in these regions and stems are often shifted.

We carried out the following tests to demonstrate how prediction quality can vary according to the homologous sequences used. We used a tmRNA alignment of 44 sequences from the tmRDB Database [24] and a RNaseP alignment of 54 sequences from the RNaseP Database [25]. Theses databases contain more sequences, but because of the time complexity of the tests, we used only a few of them. The sequences were chosen arbitrary, eliminating sequences with less than 30% of identity and sequences which are redundant. The sequences are re-aligned using ClustalW before performing the structure prediction in order to avoid other secondary structure information than ours.

We used the algorithm *P-DCfold *to predict the secondary structure of the *Escherichia coli *tmRNA and RNaseP. *P-DCfold *needed 4 homologous sequences: the number of homologous sequences used by *P-DCfold *depends on the length of the target sequence (more the target sequence is long, more homologous sequences are needed), and is at least equal to 4 sequences [13]. We then made predictions using each combination of 4 homologous sequences of the considered alignments. We calculated a quality score for each prediction according to differences from a known reference structure provided by the tmRDB Database [24] and the RNaseP Database [25]. We employed a slightly modified version of the Matthews correlation coefficient (MCC) [26] defined in [14] by Gardner and Giegerich, which measures both sensitivity (X) and selectivity (Y):

X=TPTP+FN
 MathType@MTEF@5@5@+=feaafiart1ev1aaatCvAUfKttLearuWrP9MDH5MBPbIqV92AaeXatLxBI9gBaebbnrfifHhDYfgasaacPC6xNi=xI8qiVKYPFjYdHaVhbbf9v8qqaqFr0xc9vqFj0dXdbba91qpepeI8k8fiI+fsY=rqGqVepae9pg0db9vqaiVgFr0xfr=xfr=xc9adbaqaaeGacaGaaiaabeqaaeqabiWaaaGcbaGaemiwaGLaeyypa0tcfa4aaSaaaeaacqWGubavcqWGqbauaeaacqWGubavcqWGqbaucqGHRaWkcqWGgbGrcqWGobGtaaaaaa@36CE@

Y=TPTP+(FP−ℰ)
 MathType@MTEF@5@5@+=feaafiart1ev1aaatCvAUfKttLearuWrP9MDH5MBPbIqV92AaeXatLxBI9gBaebbnrfifHhDYfgasaacPC6xNi=xI8qiVKYPFjYdHaVhbbf9v8qqaqFr0xc9vqFj0dXdbba91qpepeI8k8fiI+fsY=rqGqVepae9pg0db9vqaiVgFr0xfr=xfr=xc9adbaqaaeGacaGaaiaabeqaaeqabiWaaaGcbaGaemywaKLaeyypa0tcfa4aaSaaaeaacqWGubavcqWGqbauaeaacqWGubavcqWGqbaucqGHRaWkcqGGOaakcqWGgbGrcqWGqbaucqGHsislt0uy0HwzTfgDPnwy1egaryqtHrhAL1wy0L2yHvdaiqaacqWFWesrcqqGPaqkaaaaaa@444A@

MCC=(TP∗TN)−(FP−ℰ)∗FN(TP+(FP−ℰ))(TP+FN)(TN+(FP−ℰ))(TP+FN)
 MathType@MTEF@5@5@+=feaafiart1ev1aaatCvAUfKttLearuWrP9MDH5MBPbIqV92AaeXatLxBI9gBaebbnrfifHhDYfgasaacPC6xNi=xI8qiVKYPFjYdHaVhbbf9v8qqaqFr0xc9vqFj0dXdbba91qpepeI8k8fiI+fsY=rqGqVepae9pg0db9vqaiVgFr0xfr=xfr=xc9adbaqaaeGacaGaaiaabeqaaeqabiWaaaGcbaGaemyta0Kaem4qamKaem4qamKaeyypa0tcfa4aaSaaaeaacqGGOaakcqWGubavcqWGqbaucqGHxiIkcqWGubavcqWGobGtcqGGPaqkcqGHsislcqGGOaakcqWGgbGrcqWGqbaucqGHsislt0uy0HwzTfgDPnwy1egaryqtHrhAL1wy0L2yHvdaiqaacqWFWesrcqGGPaqkcqGHxiIkcqWGgbGrcqWGobGtaeaadaGcaaqaaiabcIcaOiabdsfaujabdcfaqjabgUcaRiabcIcaOiabdAeagjabdcfaqjabgkHiTiab=btifjabcMcaPiabcMcaPiabcIcaOiabdsfaujabdcfaqjabgUcaRiabdAeagjabd6eaojabcMcaPiabcIcaOiabdsfaujabd6eaojabgUcaRiabcIcaOiabdAeagjabdcfaqjabgkHiTiab=btifjabcMcaPiabcMcaPiabcIcaOiabdsfaujabdcfaqjabgUcaRiabdAeagjabd6eaojabcMcaPaqabaaaaaaa@704A@

where *TP *is the number of correctly predicted base pairs (true positives), *FP *the number of incorrectly predicted base pairs (false positives), *FN *the number of base pairs not found (false negatives) and *TN *the number of true negatives which is equal to (n * (*n *- 1)/2) - *TP *- *FN *- *FP*, with *n *being the length of the sequence. Because not all of the false positives are necessarily false, Gardner and Giegerich introduced the ℰ
 MathType@MTEF@5@5@+=feaafiart1ev1aaatCvAUfKttLearuWrP9MDH5MBPbIqV92AaeXatLxBI9gBaebbnrfifHhDYfgasaacPC6xNi=xH8viVGI8Gi=hEeeu0xXdbba9frFj0xb9qqpG0dXdb9aspeI8k8fiI+fsY=rqGqVepae9pg0db9vqaiVgFr0xfr=xfr=xc9adbaqaaeGacaGaaiaabeqaaeqabiWaaaGcbaWenfgDOvwBHrxAJfwnHbqeg0uy0HwzTfgDPnwy1aaceaqcfaOae8hmHueaaa@3739@ value, representing the number of false positive pairings that are not in conflict with the pairings of the reference structure. MCC ranges from -1 for extremely inaccurate predictions to 1 for very accurate ones, and is generally between 0 and 1. We set a threshold of 0.75, above which we considered a prediction to be good. Only a few percent of the possible combinations accurately predicted the structure: around one percent for tmRNA and RNaseP (Table 1). Hence, there is only one chance in a hundred of obtaining a good prediction without criteria for selecting homologous sequences.

**Table 1:** Characteristics of secondary structure predictions performed using the *P-DCfold *algorithm on a tmRNA alignment of 44 sequences and a RNaseP alignment of 54 sequences, when all possible combinations of 4 homologous sequences are considered (left) and when only combinations of 4 sequences among 10 homologous sequences initially selected by the common homology model *M*_*HC *_are considered (right).

	All sequences		*M*_*HC*_	
	tmRNA	RNaseP	tmRNA	RNaseP

Total number of predictions	123410	266699	210	210
Nb of predictions with MCC > 75	1620	1958	18	38
Average MCC	45.19	41.03	56,82	60,27
Maximum MCC	89	86	85	84
Minimum MCC	10	5	26	30

One common method to minimize the heterogeneity of prediction results is to select homologous sequences of pairwise sequence identities between 60% and 80% according to the target sequence, and to remove identical or almost identical sequences from the alignment. This is the common homology model *M*_*HC*_. We used this method to select ten homologous sequences from the alignments of the tmRNA and the RNaseP considered above. The predictions for each combination of 4 sequences from among these 10 sequences gave better prediction scores (MCC) than the predictions did using all sequences (see Table 1). The average MCC was increased from 45.19 to 56.82 for the tmRNA, and from 41.03 to 60.27 for the RNaseP. As shown in Table 1, the probability that the *M*_*HC *_selection method gives a good prediction for tmRNA was 8.5%, and it was 18% for a good prediction for RNaseP.

These results show the importance of selecting appropriate homologous sequences for efficiently predicting the secondary structure of an RNA. We have therefore designed an algorithm for selecting combinations of homologous sequences that give better prediction scores (MCC) than those obtained with the *M*_*HC *_method.

## Methods and Results

### Criteria for selecting homologous sequences

The most appropriate homologous sequences are those that have adequate variability and correct stem alignment. We need information on the substitutions by which the target sequence and the homologous sequences differ in order to evaluate these parameters. This information is calculated using substitution matrices. A substitution matrix is built for each homologous sequence: it contains all the substitution rates between this sequence and the target sequence for the four bases A, C, G and U.

#### Variability criteria

We want to select those homologous sequences that are variable enough to have compensatory mutations yet close enough to be compared with the target sequence. Hence, we defined the "adequate variability" of a homologous sequence with respect to the target sequence, depending on the percentages of identities and deletions.

The comparison step in predicting the secondary structure of an RNA by the comparative approach consists in looking for those compensatory substitutions that indicate the relevance of the stems. A stem is relevant when the number of compensatory substitutions per base exceeds a threshold *T*. Adequate variability depends on the number of homologous sequences used to predict the structure. The probability of finding compensatory mutations increases with the number of sequences used. If *N *is the number of homologous sequences used to predict the structure, the adequate identity *I *of the homologous sequences is:

I=1−TN
 MathType@MTEF@5@5@+=feaafiart1ev1aaatCvAUfKttLearuWrP9MDH5MBPbIqV92AaeXatLxBI9gBaebbnrfifHhDYfgasaacPC6xNi=xI8qiVKYPFjYdHaVhbbf9v8qqaqFr0xc9vqFj0dXdbba91qpepeI8k8fiI+fsY=rqGqVepae9pg0db9vqaiVgFr0xfr=xfr=xc9adbaqaaeGacaGaaiaabeqaaeqabiWaaaGcbaGaemysaKKaeyypa0JaeGymaeJaeyOeI0scfa4aaSaaaeaacqWGubavaeaacqWGobGtaaaaaa@3313@

The percentage of deletion was indexed to the percentage identity. This allowed us to eliminate those homologous sequences that have an abnormally high percentage of deletions compared to their percentage of identities (due to long deletions). We assume the following relation for the adequate percentage of deletion:

D=I75
 MathType@MTEF@5@5@+=feaafiart1ev1aaatCvAUfKttLearuWrP9MDH5MBPbIqV92AaeXatLxBI9gBaebbnrfifHhDYfgasaacPC6xNi=xI8qiVKYPFjYdHaVhbbf9v8qqaqFr0xc9vqFj0dXdbba91qpepeI8k8fiI+fsY=rqGqVepae9pg0db9vqaiVgFr0xfr=xfr=xc9adbaqaaeGacaGaaiaabeqaaeqabiWaaaGcbaGaemiraqKaeyypa0tcfa4aaSaaaeaacqWGjbqsaeaacqaI3aWncqaI1aqnaaaaaa@31E5@

We also discarded sequences with ambiguous or indeterminate bases.

#### Stem alignment criteria

**Figure 1:**
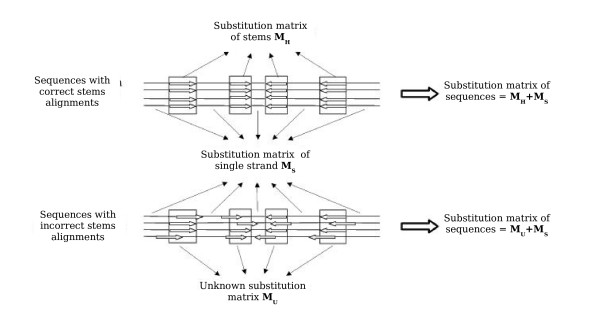
Correct and incorrect stem alignments. Single strand regions are generally correctly aligned because they are less variable, whereas the stem regions can be incorrectly aligned. Correct stem alignment results in an alternation between a stem substitution matrix (*M*_*H*_) and a single strand substitution matrix (*M*_*S*_). On the other hand, the single strand substitution matrix (*M*_*S*_) alternates with another substitution matrix (*M*_*U*_) different from the stem substitution matrix if stems are not correctly aligned.

Evolution acts to conserve the structure of an RNA molecule that is essential for its function. The sequence of a stem region is less important than the base pairing. Mutations that do not conserve the pairing disrupt the stem and can be deleterious if this stem is functionally important. On the contrary, compensatory mutations on both sides of a pair, which conserve the pairings, are not deleterious. Therefore, pairing constraints mean that not all the possible mutations are conserved by evolution in stems. The result will be a deviation of the substitution matrix in stem regions compared to single strand regions. Single strand regions are generally correctly aligned because they are less variable, whereas the stem regions can be incorrectly aligned [21,22]. Correct stem alignment results in an alternation between a stem substitution matrix *M*_*H *_and a single strand substitution matrix *M*_*S*_, and the substitution matrix of the sequence is influenced by the stem substitution matrix *M*_*H*_. On the other hand, the single strand substitution matrix *M*_*S *_alternates with another substitution matrix *M*_*U *_different from M_*H *_if stems are not correctly aligned (Figure 1).

Since *M*_*S *_is identical in correct and incorrect alignments, the difference between the two types of sequence is due solely to the alignment around and within stems, therefore to *M*_*U *_and *M*_*H*_. If we can identify *M*_*H*_, we will be able to differentiate correct alignments from incorrect alignments, and choose well-aligned homologous sequences. We must therefore identify the phenomena that influence the substitution matrices in stems and find criteria for selecting homologous sequences with correct stem alignments.

In the following are described three possible influences on stems: stability of base pairs in stems, differences between transitions and transversions, and intermediate states in double substitutions.

##### Stability of base pairs

The GC base pair is more stable than the AU base pair and the AU base pair is more stable than the GU base pair. Because of these differences in stability, GC base pairs are preferred when a stem is important for maintaining the overall structure, while GU base pairs are disadvantaged. The result is that stems are composed of a majority of GC base pairs [16]. There should therefore be a difference in the substitution matrices of stems.

**Figure 2:**
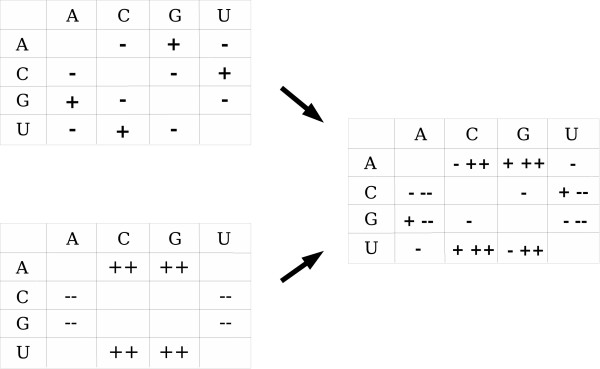
Theoretical stem substitution matrices. Left top: Stem deviation matrix due to influences of transitions/transversions and of GU intermediate state on stem substitution matrices. Left bottom: Stem deviation matrix due to influences of GC stability. Right: Stem deviation matrix due to all the influences.

Let us consider all the possible substitutions between base pairs, without the less frequent GU base pairs. We can eliminate AU ↔ UA and CG ↔ GC substitutions since they are symmetrical and do not change the substitution matrices. For the four other substitutions, if GC pairs are preferred in stems, the balance between base pairs will tend towards GC base pairs. The result will be a deviation of the nucleotide substitution rates (Figure 2, left bottom). There must therefore be more A → C and A → G substitutions than A → U substitutions, and more U → C and U → G than U → A, and fewer C → A and C → U than C → G, and fewer G → A and G → U than G → C.

Why do stems not consist entirely of GC base pairs? This is undoubtedly because stems tend towards stability, but not rigidity. Both constraints lead to a balance between the percentage of each base pair, with many GC pairs and few GU pairs.

##### Transitions versus transversions

Mutations that involve two bases of the same kind (two purines (A and G) or two pyrimidines (C and U)) are transitions (G ↔ A and C ↔ U). The others are transversions (G ↔ U, C ↔ G, U ↔ A and C ↔ A). Transitions occur more easily than transversions [23]. This phenomenon is accentuated in stems since the mutations involve base pairs. Thus, the substitution matrix in stems will be deviated positively for transitions and negatively for transversions (Figure 2 left top).

##### Stability of intermediate states

Double mutations between base pairs cannot appear simultaneously because of the low mutation rate, so they use an intermediate state (for instance AU → UU → UA). Double mutations are supported or disadvantaged depending on the stability of this intermediate state. It may be a very deleterious unpaired state or a GU pairing state that is only slightly deleterious [27]. Nevertheless, the intermediate state is rarely observed in sequence alignments [28]; this is because the intermediate state is kept rare by selection [22]. As the GU pair is the most stable and the least deleterious of the intermediate states, the double substitutions which use the GU intermediate state may occur more frequently than the others [28].

**Figure 3:**
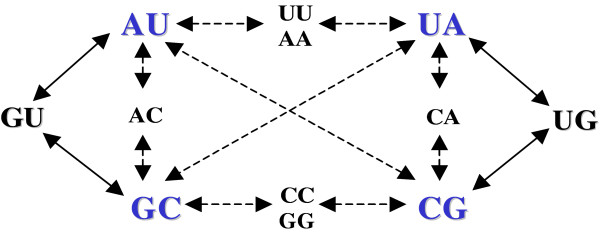
Base pair substitutions in stems. Double mutations are supported or disadvantaged depending on the stability of the intermediate state. As the GU pair is the most stable and the least deleterious of the intermediate states, the double substitutions which use the GU intermediate state (AU ↔ GC and UA ↔ CG) may occur more frequently than the others.

The double substitutions which use this intermediate state are AU ↔ GC and UA ↔ CG (Figure 3). Hence, the substitution rates of AU ↔ GC and UA ↔ CG must be increased in stems. The substitutions to and from GU (and UG) base pairs do not increase because the frequency of GU (and UG) intermediate states are kept low by selection.

As the base pair substitutions AU ↔ GC and UA ↔ CG imply substitutions A ↔ G and C ↔ U, the result must be an increase in A ↔ G and C ↔ U nucleotide substitutions over those of the other substitutions (Figure 2 left top).

##### Deviation substitution matrix summarizing all the influences in stems

Since the influence of transitions/transversions and of the GU intermediate state give the same deviation (Figure 2 left top), we cannot measure them separately. Therefore, we group them together as one influence. Considering this deviation matrix (Figure 2 left top) and the deviation matrix measuring the influence of base pair stability (Figure 2 left bottom) together, we obtain a single new deviation matrix that summarizes the three influences described above (Figure 2 right).

The influences neutralize each other for some substitutions (A → C, C → U, G → A, U → G). Since we cannot easily assign weights to each of these influences, we can measure the three influences at the same time only by comparing the squares A → G and U → C, which undergo all the influences positively, and the squares C → A and G → U, which undergo all the influences negatively. If we want to measure each influence separately, we must compare substitutions that change only for one influence:

• The influence of GC stability is measured by comparing A → C with C → A, U → C with C → U, A → G with G → A and U → G with G → U, since the difference is due only to the influence of GC stability in these corresponding matrix squares.

• The influence of the GU intermediate state and the influence of transitions/transversions are measured by comparing substitutions between A → G and A → C, U → C and U → G, C → U and C → A, G → A and G → U.

#### Existing evolutionary models under structure constraints

Many models of evolution under secondary structure constraints use the idea of a GU intermediate state. In [29], these models have been compared using maximum likelihood methods and sequences of the small subunit ribosomal RNA. They conclude that the Higgs model [30] is the best for this set of RNA sequences. The base pair frequencies given in [30] confirm our idea of base pair stability: GC base pair is the most frequent. Also, the base pair substitution rates given in [30] confirm the idea of intermediate state stability. We used the best base pair mutabilities and frequencies obtained by Higgs to verify the deviation matrix in Figure 2. We computed the matrix of nucleotide substitutions rates from the Higgs values (obtained by Higgs [30]) as follows:

P(i→j)=P(ik)×∑k,lP(ik→jl)+P(ki)×∑k,lP(ki→lj)
 MathType@MTEF@5@5@+=feaafiart1ev1aaatCvAUfKttLearuWrP9MDH5MBPbIqV92AaeXatLxBI9gBaebbnrfifHhDYfgasaacPC6xNi=xI8qiVKYPFjYdHaVhbbf9v8qqaqFr0xc9vqFj0dXdbba91qpepeI8k8fiI+fsY=rqGqVepae9pg0db9vqaiVgFr0xfr=xfr=xc9adbaqaaeGacaGaaiaabeqaaeqabiWaaaGcbaGaemiuaa1aaSbaaSqaaiabcIcaOiabdMgaPjabgkziUkabdQgaQjabcMcaPaqabaGccqGH9aqpcqWGqbaudaWgaaWcbaGaeiikaGIaemyAaKMaem4AaSMaeiykaKcabeaakiabgEna0oaaqafabaGaemiuaa1aaSbaaSqaaiabcIcaOiabdMgaPjabdUgaRjabgkziUkabdQgaQjabdYgaSjabcMcaPaqabaGccqGHRaWkcqWGqbaudaWgaaWcbaGaeiikaGIaem4AaSMaemyAaKMaeiykaKcabeaaaeaacqWGRbWAcqGGSaalcqWGSbaBaeqaniabggHiLdGccqGHxdaTdaaeqbqaaiabdcfaqnaaBaaaleaacqGGOaakcqWGRbWAcqWGPbqAcqGHsgIRcqWGSbaBcqWGQbGAcqGGPaqkaeqaaaqaaiabdUgaRjabcYcaSiabdYgaSbqab0GaeyyeIuoaaaa@65D5@

with *P*_(*ik*) _the frequency of the base pair *ik *and *P*_(*ik*→*jl*) _the probability of a mutation from a base pair *ik *to a base pair *jl*.

The obtained matrix (Table 2) confirms the deviation matrix in Figure 2:. *P*_*A*→*G *_> *P*_*A*→*U*_, *P*_*C*→*U *_> *P*_*C*→*G *_> *P*_*C*→*A*_, *P*_*G*→*A *_> *P*_*G*→*C *_> *P*_*G*→*U *_and *P*_*U*→*C *_> *P*_*U*→*G *_> *P*_*U*→*A*_.

**Table 2:** Nucleotide substitution rates calculated with the Higgs model parameters [30].

	A	C	G	U
A		0.0201	0.1911	0.0642
C	0.0185		0.1194	0.1948
G	0.1915	0.1180		0.0871
U	0.0641	0.1947	0.0873	

#### Existing base pair substitution matrices

The RIBOSUM85-60 matrix obtained by Klein and Eddy [31] confirms our models. This matrix gives the log-odds ratio for a given base pair substitution relative to background nucleotide frequencies. It confirms the idea of a deviation of the substitutions due to base pair stability. Adding together the scores of the matrix that include the pairs GC and CG gives a score of 26.12. This score is 22.14 for the pairs AU and UA and 5.48 for the pairs GU and UG. Thus the GC (and CG) pairs are more attractive than the other pairs (AU, UA, GU and UG). Hence, substitutions that give GC (and CG) pairs are more abundant than the others. The matrix also confirms the stability of GU intermediate state. The frequencies of AU ↔ GC and UA ↔ CG substitutions (which uses the GU intermediate state) are higher than the frequencies of the other substitutions. The substitutions GU ↔ CG, GU ↔ UA, UG ↔ GC, UG ↔ AU have negative scores.

### The algorithm SSCA for selecting homologous sequences

The algorithm *SSCA *is based on a selection model, ℳ
 MathType@MTEF@5@5@+=feaafiart1ev1aaatCvAUfKttLearuWrP9MDH5MBPbIqV92AaeXatLxBI9gBaebbnrfifHhDYfgasaacPC6xNi=xH8viVGI8Gi=hEeeu0xXdbba9frFj0xb9qqpG0dXdb9aspeI8k8fiI+fsY=rqGqVepae9pg0db9vqaiVgFr0xfr=xfr=xc9adbaqaaeGacaGaaiaabeqaaeqabiWaaaGcbaWenfgDOvwBHrxAJfwnHbqeg0uy0HwzTfgDPnwy1aaceaqcfaOae83mH0eaaa@3744@, that represents constraints on the substitution matrices of the homologous sequences towards the target sequence. These constraints model an ideal homologous sequence having adequate variability and correct stems alignment.

*SSCA *calculates the substitution matrix for each homologous sequence with respect to the target sequence. A usefulness score is then calculated for the sequence according to the model ℳ
 MathType@MTEF@5@5@+=feaafiart1ev1aaatCvAUfKttLearuWrP9MDH5MBPbIqV92AaeXatLxBI9gBaebbnrfifHhDYfgasaacPC6xNi=xH8viVGI8Gi=hEeeu0xXdbba9frFj0xb9qqpG0dXdb9aspeI8k8fiI+fsY=rqGqVepae9pg0db9vqaiVgFr0xfr=xfr=xc9adbaqaaeGacaGaaiaabeqaaeqabiWaaaGcbaWenfgDOvwBHrxAJfwnHbqeg0uy0HwzTfgDPnwy1aaceaqcfaOae83mH0eaaa@3744@ and its substitution matrix. The sequences closest to the ideal sequence (modeled by ℳ
 MathType@MTEF@5@5@+=feaafiart1ev1aaatCvAUfKttLearuWrP9MDH5MBPbIqV92AaeXatLxBI9gBaebbnrfifHhDYfgasaacPC6xNi=xH8viVGI8Gi=hEeeu0xXdbba9frFj0xb9qqpG0dXdb9aspeI8k8fiI+fsY=rqGqVepae9pg0db9vqaiVgFr0xfr=xfr=xc9adbaqaaeGacaGaaiaabeqaaeqabiWaaaGcbaWenfgDOvwBHrxAJfwnHbqeg0uy0HwzTfgDPnwy1aaceaqcfaOae83mH0eaaa@3744@) are the most useful for predicting the secondary structure (Table 3).

**Table 3:** Algorithm *SSCA*

**Algorithm ***SSCA *(*S*_*t*_: target sequence, *Al*: homologous sequences alignment)
**Begin**
-Build a model ℳ MathType@MTEF@5@5@+=feaafiart1ev1aaatCvAUfKttLearuWrP9MDH5MBPbIqV92AaeXatLxBI9gBaebbnrfifHhDYfgasaacPC6xNi=xH8viVGI8Gi=hEeeu0xXdbba9frFj0xb9qqpG0dXdb9aspeI8k8fiI+fsY=rqGqVepae9pg0db9vqaiVgFr0xfr=xfr=xc9adbaqaaeGacaGaaiaabeqaaeqabiWaaaGcbaWenfgDOvwBHrxAJfwnHbqeg0uy0HwzTfgDPnwy1aaceaqcfaOae83mH0eaaa@3744@ according to constraints
-For each homologous sequence *S*_*i *_of *Al*
-Calculate the substitution matrix *M*_*i *_between *S*_*t *_and *S*_*i*_
-Calculate a score for *S*_*i *_according to the constraints of the model ℳ MathType@MTEF@5@5@+=feaafiart1ev1aaatCvAUfKttLearuWrP9MDH5MBPbIqV92AaeXatLxBI9gBaebbnrfifHhDYfgasaacPC6xNi=xH8viVGI8Gi=hEeeu0xXdbba9frFj0xb9qqpG0dXdb9aspeI8k8fiI+fsY=rqGqVepae9pg0db9vqaiVgFr0xfr=xfr=xc9adbaqaaeGacaGaaiaabeqaaeqabiWaaaGcbaWenfgDOvwBHrxAJfwnHbqeg0uy0HwzTfgDPnwy1aaceaqcfaOae83mH0eaaa@3744@ and to the substitution matrix *M*_*i*_
-Classify the sequences *S*_*i *_according to their score
**End**

The model ℳ
 MathType@MTEF@5@5@+=feaafiart1ev1aaatCvAUfKttLearuWrP9MDH5MBPbIqV92AaeXatLxBI9gBaebbnrfifHhDYfgasaacPC6xNi=xH8viVGI8Gi=hEeeu0xXdbba9frFj0xb9qqpG0dXdb9aspeI8k8fiI+fsY=rqGqVepae9pg0db9vqaiVgFr0xfr=xfr=xc9adbaqaaeGacaGaaiaabeqaaeqabiWaaaGcbaWenfgDOvwBHrxAJfwnHbqeg0uy0HwzTfgDPnwy1aaceaqcfaOae83mH0eaaa@3744@ has two parts: one concerns the sequence variability and the other the stem alignment.

#### Homologous sequence selection constraints

##### Constraints for selecting homologous sequences according to their variabilities

The constraints on the selection of homologous sequences according to adequate variability are:

{C1=|(x→x)−I|x∈{A,C,G,U}C2=|(x→′−′)−D|x∈{A,C,G,U}C3=|(x→′N′)|x∈{A,C,G,U}SV=C1+C2+C3
 MathType@MTEF@5@5@+=feaafiart1ev1aaatCvAUfKttLearuWrP9MDH5MBPbIqV92AaeXatLxBI9gBaebbnrfifHhDYfgasaacPC6xNi=xI8qiVKYPFjYdHaVhbbf9v8qqaqFr0xc9vqFj0dXdbba91qpepeI8k8fiI+fsY=rqGqVepae9pg0db9vqaiVgFr0xfr=xfr=xc9adbaqaaeGacaGaaiaabeqaaeqabiWaaaGcbaWaaiqabeaafaqaaeabcaaaaeaacqWGdbWqcqaIXaqmcqGH9aqpdaabdaqaaiabcIcaOiabdIha4jabgkziUkabdIha4jabcMcaPiabgkHiTiabdMeajbGaay5bSlaawIa7aaqaaiabdIha4jabgIGiolabcUha7jabdgeabjabcYcaSiabdoeadjabcYcaSiabdEeahjabcYcaSiabdwfavjabc2ha9bqaaiabdoeadjabikdaYiabg2da9maaemaabaGaeiikaGIaemiEaGNafyOKH4QbauaacuGHsislgaqbaiabcMcaPiabgkHiTiabdseaebGaay5bSlaawIa7aaqaaiabdIha4jabgIGiolabcUha7jabdgeabjabcYcaSiabdoeadjabcYcaSiabdEeahjabcYcaSiabdwfavjabc2ha9bqaaiabdoeadjabiodaZiabg2da9maaemaabaGaeiikaGIaemiEaGNafyOKH4QbauaacuWGobGtgaqbaiabcMcaPaGaay5bSlaawIa7aaqaaiabdIha4jabgIGiolabcUha7jabdgeabjabcYcaSiabdoeadjabcYcaSiabdEeahjabcYcaSiabdwfavjabc2ha9bqaaiabdofatnaaBaaaleaacqWGwbGvaeqaaOGaeyypa0Jaem4qamKaeGymaeJaey4kaSIaem4qamKaeGOmaiJaey4kaSIaem4qamKaeG4mamdabaaaaaGaay5Eaaaaaa@893F@

Given a homologous sequence, constraint *C*1 measures the difference between the conservation rate and the adequate identity rate *I *described in the equation (1) (given above). Constraint *C*2 measures the difference between the deletion rate and the adequate deletion rate *D *described in the equation (2). *C*3 measures the rate of ambiguous or indeterminate bases ('*N*').

Homologous sequences with adequate variability are then selected by minimizing the sum *S*_*V *_of the constraints *C*1, *C*2 and *C*3.

##### Constraints for selecting homologous sequences according to their stem alignment

We have proposed two methods for building the second part of the model and selecting homologous sequences according to their stem alignments. These methods emphasize the three influences in stem regions described above. Each method provides a model that can be used in our algorithm *SSCA*:

The first method measures the influence of the GU intermediate state and differences between transitions and transversions. A → G is compared to A → C, U → C to U → G, C → U to C → A and G → A to G → U, applying constraints on squares of the substitution matrices for each homologous sequence:

{C4=(A→G)−(A→C)C5=(U→C)−(U→G)C6=(C→U)−(C→A)C7=(G→A)−(G→U)SA1=C4+C5+C6+C7
 MathType@MTEF@5@5@+=feaafiart1ev1aaatCvAUfKttLearuWrP9MDH5MBPbIqV92AaeXatLxBI9gBaebbnrfifHhDYfgasaacPC6xNi=xI8qiVKYPFjYdHaVhbbf9v8qqaqFr0xc9vqFj0dXdbba91qpepeI8k8fiI+fsY=rqGqVepae9pg0db9vqaiVgFr0xfr=xfr=xc9adbaqaaeGacaGaaiaabeqaaeqabiWaaaGcbaWaaiqabeaafaqadeqbbaaaaeaacqWGdbWqcqaI0aancqGH9aqpcqGGOaakcqWGbbqqcqGHsgIRcqWGhbWrcqGGPaqkcqGHsislcqGGOaakcqWGbbqqcqGHsgIRcqWGdbWqcqGGPaqkaeaacqWGdbWqcqaI1aqncqGH9aqpcqGGOaakcqWGvbqvcqGHsgIRcqWGdbWqcqGGPaqkcqGHsislcqGGOaakcqWGvbqvcqGHsgIRcqWGhbWrcqGGPaqkaeaacqWGdbWqcqaI2aGncqGH9aqpcqGGOaakcqWGdbWqcqGHsgIRcqWGvbqvcqGGPaqkcqGHsislcqGGOaakcqWGdbWqcqGHsgIRcqWGbbqqcqGGPaqkaeaacqWGdbWqcqaI3aWncqGH9aqpcqGGOaakcqWGhbWrcqGHsgIRcqWGbbqqcqGGPaqkcqGHsislcqGGOaakcqWGhbWrcqGHsgIRcqWGvbqvcqGGPaqkaeaacqWGtbWudaWgaaWcbaGaemyqaeKaeGymaedabeaakiabg2da9iabdoeadjabisda0iabgUcaRiabdoeadjabiwda1iabgUcaRiabdoeadjabiAda2iabgUcaRiabdoeadjabiEda3aaaaiaawUhaaaaa@7AF1@

Constraints C4, C5, C6 and C7 measure the differences between the substitution rates A → G, U → C, C → U and G → A and the substitution rates A → C, U → G, C → A and G → U. The score *S*_*A*1 _is then maximized to select sequences that are greatly influenced by the GU intermediate state.

• The second method measures GC stability. It compares A → C with C → A, U → C with C → U, A → G with G → A and U → G with G → U, using the constraints:

{C4=(A→C)−(C→A)C5=(A→G)−(G→A)C6=(U→C)−(C→U)C7=(U→G)−(G→U)SA2=C4+C5+C6+C7
 MathType@MTEF@5@5@+=feaafiart1ev1aaatCvAUfKttLearuWrP9MDH5MBPbIqV92AaeXatLxBI9gBaebbnrfifHhDYfgasaacPC6xNi=xI8qiVKYPFjYdHaVhbbf9v8qqaqFr0xc9vqFj0dXdbba91qpepeI8k8fiI+fsY=rqGqVepae9pg0db9vqaiVgFr0xfr=xfr=xc9adbaqaaeGacaGaaiaabeqaaeqabiWaaaGcbaWaaiqabeaafaqadeqbbaaaaeaacqWGdbWqcqaI0aancqGH9aqpcqGGOaakcqWGbbqqcqGHsgIRcqWGdbWqcqGGPaqkcqGHsislcqGGOaakcqWGdbWqcqGHsgIRcqWGbbqqcqGGPaqkaeaacqWGdbWqcqaI1aqncqGH9aqpcqGGOaakcqWGbbqqcqGHsgIRcqWGhbWrcqGGPaqkcqGHsislcqGGOaakcqWGhbWrcqGHsgIRcqWGbbqqcqGGPaqkaeaacqWGdbWqcqaI2aGncqGH9aqpcqGGOaakcqWGvbqvcqGHsgIRcqWGdbWqcqGGPaqkcqGHsislcqGGOaakcqWGdbWqcqGHsgIRcqWGvbqvcqGGPaqkaeaacqWGdbWqcqaI3aWncqGH9aqpcqGGOaakcqWGvbqvcqGHsgIRcqWGhbWrcqGGPaqkcqGHsislcqGGOaakcqWGhbWrcqGHsgIRcqWGvbqvcqGGPaqkaeaacqWGtbWudaWgaaWcbaGaemyqaeKaeGOmaidabeaakiabg2da9iabdoeadjabisda0iabgUcaRiabdoeadjabiwda1iabgUcaRiabdoeadjabiAda2iabgUcaRiabdoeadjabiEda3aaaaiaawUhaaaaa@7AF3@

Constraints C4, C5, C6 and C7 measure the differences between the substitution rates A → C, A → G, U → C and U → G and the substitution rates C → A, G → A, C → U and G → U. The score *S*_*A*2 _is then maximized to select sequences greatly influenced by the GC intermediate state.

#### Models for sequence selection

Each method of calculating the second part of the model (stem alignment) is combined with the method of calculating the first part of the model (variability). We thus obtained two models for selecting homologous sequences, each of which can be used to calculate a score for each homologous sequence:

• Model ℳGU
 MathType@MTEF@5@5@+=feaafiart1ev1aaatCvAUfKttLearuWrP9MDH5MBPbIqV92AaeXatLxBI9gBaebbnrfifHhDYfgasaacPC6xNi=xH8viVGI8Gi=hEeeu0xXdbba9frFj0xb9qqpG0dXdb9aspeI8k8fiI+fsY=rqGqVepae9pg0db9vqaiVgFr0xfr=xfr=xc9adbaqaaeGacaGaaiaabeqaaeqabiWaaaGcbaWenfgDOvwBHrxAJfwnHbqeg0uy0HwzTfgDPnwy1aaceaGae83mH00aaSbaaSqaaiab=zq8hjab=rr8vbqabaaaaa@3A84@ with a calculated score of *S*_*GU *_= *S*_*V *_- *S*_*A*1_

• Model ℳGC
 MathType@MTEF@5@5@+=feaafiart1ev1aaatCvAUfeBSjuyZL2yd9gzLbvyNv2CaerbwvMCKfMBHbqedmvETj2BSbqee0evGueE0jxyaibaieYdOi=BI8qipeYdI8qiW7rqqrFfpeea0xe9LqFf0xc9q8qqaqFn0dXdHiVcFbIOFHK8Feei0lXdar=Jb9qqFfeaYRXxe9vr0=vr0=LqpWqaaeaabiGaaiaacaqabeaabeqacmaaaOqaamrtHrhAL1wy0L2yHvtyaeHbnfgDOvwBHrxAJfwnaGabaiab=ntinnaaBaaaleaacqWFge=rcqWFce=qaeqaaaaa@3BB7@ with a calculated score of *S*_*GC *_= *S*_*V *_- *S*_*A*2_

Another model, which is a combination of the two above, provides a measure of the combined influences of GC pairing stability and of the GU intermediate state:

• Model ℳGC+GU
 MathType@MTEF@5@5@+=feaafiart1ev1aaatCvAUfKttLearuWrP9MDH5MBPbIqV92AaeXatLxBI9gBaebbnrfifHhDYfgasaacPC6xNi=xH8viVGI8Gi=hEeeu0xXdbba9frFj0xb9qqpG0dXdb9aspeI8k8fiI+fsY=rqGqVepae9pg0db9vqaiVgFr0xfr=xfr=xc9adbaqaaeGacaGaaiaabeqaaeqabiWaaaGcbaWenfgDOvwBHrxAJfwnHbqeg0uy0HwzTfgDPnwy1aaceaGae83mH00aaSbaaSqaaiab=zq8hjab=jq8djabgUcaRiab=zq8hjab=rr8vbqabaaaaa@3EE4@ with a calculated score of *S*_*GC*+*GU *_= *S*_*GC *_+ *S*_*GU*_

The homologous sequences to be used to predict the structure of the target sequence are selected according to their *S*_*GU*_, *S*_*GC *_or *S*_*GC*+*GU *_scores. The most suitable homologous sequences for the comparative approach have the lowest scores (since *S*_*A*1 _and *S*_*A*2 _are maximized and *S*_*V *_minimized). The algorithm *SSCA *can be used using each of the three models ℳGU
 MathType@MTEF@5@5@+=feaafiart1ev1aaatCvAUfKttLearuWrP9MDH5MBPbIqV92AaeXatLxBI9gBaebbnrfifHhDYfgasaacPC6xNi=xH8viVGI8Gi=hEeeu0xXdbba9frFj0xb9qqpG0dXdb9aspeI8k8fiI+fsY=rqGqVepae9pg0db9vqaiVgFr0xfr=xfr=xc9adbaqaaeGacaGaaiaabeqaaeqabiWaaaGcbaWenfgDOvwBHrxAJfwnHbqeg0uy0HwzTfgDPnwy1aaceaGae83mH00aaSbaaSqaaiab=zq8hjab=rr8vbqabaaaaa@3A84@, ℳGC
 MathType@MTEF@5@5@+=feaafiart1ev1aaatCvAUfeBSjuyZL2yd9gzLbvyNv2CaerbwvMCKfMBHbqedmvETj2BSbqee0evGueE0jxyaibaieYdOi=BI8qipeYdI8qiW7rqqrFfpeea0xe9LqFf0xc9q8qqaqFn0dXdHiVcFbIOFHK8Feei0lXdar=Jb9qqFfeaYRXxe9vr0=vr0=LqpWqaaeaabiGaaiaacaqabeaabeqacmaaaOqaamrtHrhAL1wy0L2yHvtyaeHbnfgDOvwBHrxAJfwnaGabaiab=ntinnaaBaaaleaacqWFge=rcqWFce=qaeqaaaaa@3BB7@ and ℳGC+GU
 MathType@MTEF@5@5@+=feaafiart1ev1aaatCvAUfKttLearuWrP9MDH5MBPbIqV92AaeXatLxBI9gBaebbnrfifHhDYfgasaacPC6xNi=xH8viVGI8Gi=hEeeu0xXdbba9frFj0xb9qqpG0dXdb9aspeI8k8fiI+fsY=rqGqVepae9pg0db9vqaiVgFr0xfr=xfr=xc9adbaqaaeGacaGaaiaabeqaaeqabiWaaaGcbaWenfgDOvwBHrxAJfwnHbqeg0uy0HwzTfgDPnwy1aaceaGae83mH00aaSbaaSqaaiab=zq8hjab=jq8djabgUcaRiab=zq8hjab=rr8vbqabaaaaa@3EE4@.

### Results

The *SSCA *algorithm was tested on several RNA sequence alignments: tmRNA, RNaseP, SRP RNA, U1 RNA and 5S RNA. It was initially tested with the *P-DCfold *algorithm [13] for predicting the secondary structure, then with the *RNAalifold *algorithm [16].

#### Results obtained using the P-DCfold algorithm

The RNAs tmRNA, RNaseP, SRP RNA, U1 RNA and 5S RNA are between 80 and 380 nucleotides long. *P-DCfold *needed four homologous sequences to predict the secondary structure of each of these RNAs. The *SSCA *algorithm was then used to select the four most suitable homologous sequences.

The following procedure was used to test and compare the three models of *SSCA *for each target sequence:

1. We predicted the structure of the target sequence with *P-DCfold *using each possible combination of the four homologous sequences. Since we know the secondary structure, we calculated and attributed the MCC scores to each prediction.

2. The algorithm *SSCA *was used to classify the homologous sequences according to scores obtained with each of the three models ℳGU
 MathType@MTEF@5@5@+=feaafiart1ev1aaatCvAUfKttLearuWrP9MDH5MBPbIqV92AaeXatLxBI9gBaebbnrfifHhDYfgasaacPC6xNi=xH8viVGI8Gi=hEeeu0xXdbba9frFj0xb9qqpG0dXdb9aspeI8k8fiI+fsY=rqGqVepae9pg0db9vqaiVgFr0xfr=xfr=xc9adbaqaaeGacaGaaiaabeqaaeqabiWaaaGcbaWenfgDOvwBHrxAJfwnHbqeg0uy0HwzTfgDPnwy1aaceaGae83mH00aaSbaaSqaaiab=zq8hjab=rr8vbqabaaaaa@3A84@, ℳGC
 MathType@MTEF@5@5@+=feaafiart1ev1aaatCvAUfeBSjuyZL2yd9gzLbvyNv2CaerbwvMCKfMBHbqedmvETj2BSbqee0evGueE0jxyaibaieYdOi=BI8qipeYdI8qiW7rqqrFfpeea0xe9LqFf0xc9q8qqaqFn0dXdHiVcFbIOFHK8Feei0lXdar=Jb9qqFfeaYRXxe9vr0=vr0=LqpWqaaeaabiGaaiaacaqabeaabeqacmaaaOqaamrtHrhAL1wy0L2yHvtyaeHbnfgDOvwBHrxAJfwnaGabaiab=ntinnaaBaaaleaacqWFge=rcqWFce=qaeqaaaaa@3BB7@ and ℳGC+GU
 MathType@MTEF@5@5@+=feaafiart1ev1aaatCvAUfKttLearuWrP9MDH5MBPbIqV92AaeXatLxBI9gBaebbnrfifHhDYfgasaacPC6xNi=xH8viVGI8Gi=hEeeu0xXdbba9frFj0xb9qqpG0dXdb9aspeI8k8fiI+fsY=rqGqVepae9pg0db9vqaiVgFr0xfr=xfr=xc9adbaqaaeGacaGaaiaabeqaaeqabiWaaaGcbaWenfgDOvwBHrxAJfwnHbqeg0uy0HwzTfgDPnwy1aaceaGae83mH00aaSbaaSqaaiab=zq8hjab=jq8djabgUcaRiab=zq8hjab=rr8vbqabaaaaa@3EE4@. We also classified them using the common homology model *M*_*HC*_.

3. In order to have a big enough sample of predictions to draw conclusions, the ten best homologous sequences for each classification were selected and each possible combination of four homologous sequences was tested using *P-DCfold*. MCC scores were calculated for the resulting 210 predictions.

##### Results for Escherichia coli tmRNA

An alignment of 44 sequences and a reference structure from the tmRDB Database [24] were used to predict the secondary structure of *Escherichia coli *tmRNA.

The four models *M*_*HC*_, ℳGU
 MathType@MTEF@5@5@+=feaafiart1ev1aaatCvAUfKttLearuWrP9MDH5MBPbIqV92AaeXatLxBI9gBaebbnrfifHhDYfgasaacPC6xNi=xH8viVGI8Gi=hEeeu0xXdbba9frFj0xb9qqpG0dXdb9aspeI8k8fiI+fsY=rqGqVepae9pg0db9vqaiVgFr0xfr=xfr=xc9adbaqaaeGacaGaaiaabeqaaeqabiWaaaGcbaWenfgDOvwBHrxAJfwnHbqeg0uy0HwzTfgDPnwy1aaceaGae83mH00aaSbaaSqaaiab=zq8hjab=rr8vbqabaaaaa@3A84@, ℳGC
 MathType@MTEF@5@5@+=feaafiart1ev1aaatCvAUfeBSjuyZL2yd9gzLbvyNv2CaerbwvMCKfMBHbqedmvETj2BSbqee0evGueE0jxyaibaieYdOi=BI8qipeYdI8qiW7rqqrFfpeea0xe9LqFf0xc9q8qqaqFn0dXdHiVcFbIOFHK8Feei0lXdar=Jb9qqFfeaYRXxe9vr0=vr0=LqpWqaaeaabiGaaiaacaqabeaabeqacmaaaOqaamrtHrhAL1wy0L2yHvtyaeHbnfgDOvwBHrxAJfwnaGabaiab=ntinnaaBaaaleaacqWFge=rcqWFce=qaeqaaaaa@3BB7@ and ℳGC+GU
 MathType@MTEF@5@5@+=feaafiart1ev1aaatCvAUfKttLearuWrP9MDH5MBPbIqV92AaeXatLxBI9gBaebbnrfifHhDYfgasaacPC6xNi=xH8viVGI8Gi=hEeeu0xXdbba9frFj0xb9qqpG0dXdb9aspeI8k8fiI+fsY=rqGqVepae9pg0db9vqaiVgFr0xfr=xfr=xc9adbaqaaeGacaGaaiaabeqaaeqabiWaaaGcbaWenfgDOvwBHrxAJfwnHbqeg0uy0HwzTfgDPnwy1aaceaGae83mH00aaSbaaSqaaiab=zq8hjab=jq8djabgUcaRiab=zq8hjab=rr8vbqabaaaaa@3EE4@ provided higher MCC scores than the ones obtained when using all the sequences (Table 4). The best results were obtained with ℳGC
 MathType@MTEF@5@5@+=feaafiart1ev1aaatCvAUfeBSjuyZL2yd9gzLbvyNv2CaerbwvMCKfMBHbqedmvETj2BSbqee0evGueE0jxyaibaieYdOi=BI8qipeYdI8qiW7rqqrFfpeea0xe9LqFf0xc9q8qqaqFn0dXdHiVcFbIOFHK8Feei0lXdar=Jb9qqFfeaYRXxe9vr0=vr0=LqpWqaaeaabiGaaiaacaqabeaabeqacmaaaOqaamrtHrhAL1wy0L2yHvtyaeHbnfgDOvwBHrxAJfwnaGabaiab=ntinnaaBaaaleaacqWFge=rcqWFce=qaeqaaaaa@3BB7@ and ℳGC+GU
 MathType@MTEF@5@5@+=feaafiart1ev1aaatCvAUfKttLearuWrP9MDH5MBPbIqV92AaeXatLxBI9gBaebbnrfifHhDYfgasaacPC6xNi=xH8viVGI8Gi=hEeeu0xXdbba9frFj0xb9qqpG0dXdb9aspeI8k8fiI+fsY=rqGqVepae9pg0db9vqaiVgFr0xfr=xfr=xc9adbaqaaeGacaGaaiaabeqaaeqabiWaaaGcbaWenfgDOvwBHrxAJfwnHbqeg0uy0HwzTfgDPnwy1aaceaGae83mH00aaSbaaSqaaiab=zq8hjab=jq8djabgUcaRiab=zq8hjab=rr8vbqabaaaaa@3EE4@: the average MCC was around 67.5 instead of 45.19 in the case of using all sequences. The results obtained with the three models, ℳGU
 MathType@MTEF@5@5@+=feaafiart1ev1aaatCvAUfKttLearuWrP9MDH5MBPbIqV92AaeXatLxBI9gBaebbnrfifHhDYfgasaacPC6xNi=xH8viVGI8Gi=hEeeu0xXdbba9frFj0xb9qqpG0dXdb9aspeI8k8fiI+fsY=rqGqVepae9pg0db9vqaiVgFr0xfr=xfr=xc9adbaqaaeGacaGaaiaabeqaaeqabiWaaaGcbaWenfgDOvwBHrxAJfwnHbqeg0uy0HwzTfgDPnwy1aaceaGae83mH00aaSbaaSqaaiab=zq8hjab=rr8vbqabaaaaa@3A84@ (average MCC of 63.38), ℳGC
 MathType@MTEF@5@5@+=feaafiart1ev1aaatCvAUfeBSjuyZL2yd9gzLbvyNv2CaerbwvMCKfMBHbqedmvETj2BSbqee0evGueE0jxyaibaieYdOi=BI8qipeYdI8qiW7rqqrFfpeea0xe9LqFf0xc9q8qqaqFn0dXdHiVcFbIOFHK8Feei0lXdar=Jb9qqFfeaYRXxe9vr0=vr0=LqpWqaaeaabiGaaiaacaqabeaabeqacmaaaOqaamrtHrhAL1wy0L2yHvtyaeHbnfgDOvwBHrxAJfwnaGabaiab=ntinnaaBaaaleaacqWFge=rcqWFce=qaeqaaaaa@3BB7@ (average MCC of 67.45) and ℳGC+GU
 MathType@MTEF@5@5@+=feaafiart1ev1aaatCvAUfKttLearuWrP9MDH5MBPbIqV92AaeXatLxBI9gBaebbnrfifHhDYfgasaacPC6xNi=xH8viVGI8Gi=hEeeu0xXdbba9frFj0xb9qqpG0dXdb9aspeI8k8fiI+fsY=rqGqVepae9pg0db9vqaiVgFr0xfr=xfr=xc9adbaqaaeGacaGaaiaabeqaaeqabiWaaaGcbaWenfgDOvwBHrxAJfwnHbqeg0uy0HwzTfgDPnwy1aaceaGae83mH00aaSbaaSqaaiab=zq8hjab=jq8djabgUcaRiab=zq8hjab=rr8vbqabaaaaa@3EE4@ (average MCC of 67.66) were better than those obtained with the model *M*_*HC *_(average MCC of 56.82).

**Table 4:** MCC distributions (Average MCC, Maximum MCC and Minimum MCC) of tmRNA and RNaseP secondary structure predictions done with the *P-DCfold *algorithm using all sequences and using different homologous sequence selection models (*M*_*HC*_, ℳGU
 MathType@MTEF@5@5@+=feaafiart1ev1aaatCvAUfKttLearuWrP9MDH5MBPbIqV92AaeXatLxBI9gBaebbnrfifHhDYfgasaacPC6xNi=xH8viVGI8Gi=hEeeu0xXdbba9frFj0xb9qqpG0dXdb9aspeI8k8fiI+fsY=rqGqVepae9pg0db9vqaiVgFr0xfr=xfr=xc9adbaqaaeGacaGaaiaabeqaaeqabiWaaaGcbaWenfgDOvwBHrxAJfwnHbqeg0uy0HwzTfgDPnwy1aaceaGae83mH00aaSbaaSqaaiab=zq8hjab=rr8vbqabaaaaa@3A84@, ℳGC
 MathType@MTEF@5@5@+=feaafiart1ev1aaatCvAUfeBSjuyZL2yd9gzLbvyNv2CaerbwvMCKfMBHbqedmvETj2BSbqee0evGueE0jxyaibaieYdOi=BI8qipeYdI8qiW7rqqrFfpeea0xe9LqFf0xc9q8qqaqFn0dXdHiVcFbIOFHK8Feei0lXdar=Jb9qqFfeaYRXxe9vr0=vr0=LqpWqaaeaabiGaaiaacaqabeaabeqacmaaaOqaamrtHrhAL1wy0L2yHvtyaeHbnfgDOvwBHrxAJfwnaGabaiab=ntinnaaBaaaleaacqWFge=rcqWFce=qaeqaaaaa@3BB7@ and ℳGC+GU
 MathType@MTEF@5@5@+=feaafiart1ev1aaatCvAUfKttLearuWrP9MDH5MBPbIqV92AaeXatLxBI9gBaebbnrfifHhDYfgasaacPC6xNi=xH8viVGI8Gi=hEeeu0xXdbba9frFj0xb9qqpG0dXdb9aspeI8k8fiI+fsY=rqGqVepae9pg0db9vqaiVgFr0xfr=xfr=xc9adbaqaaeGacaGaaiaabeqaaeqabiWaaaGcbaWenfgDOvwBHrxAJfwnHbqeg0uy0HwzTfgDPnwy1aaceaGae83mH00aaSbaaSqaaiab=zq8hjab=jq8djabgUcaRiab=zq8hjab=rr8vbqabaaaaa@3EE4@). The percentage of predictions with MCC > 75 are also given.

	tmRNA					RNAseP				
	All	*M*_*HC*_	ℳGU MathType@MTEF@5@5@+=feaafiart1ev1aaatCvAUfKttLearuWrP9MDH5MBPbIqV92AaeXatLxBI9gBaebbnrfifHhDYfgasaacPC6xNi=xH8viVGI8Gi=hEeeu0xXdbba9frFj0xb9qqpG0dXdb9aspeI8k8fiI+fsY=rqGqVepae9pg0db9vqaiVgFr0xfr=xfr=xc9adbaqaaeGacaGaaiaabeqaaeqabiWaaaGcbaWenfgDOvwBHrxAJfwnHbqeg0uy0HwzTfgDPnwy1aaceaGae83mH00aaSbaaSqaaiab=zq8hjab=rr8vbqabaaaaa@3A84@	ℳGC MathType@MTEF@5@5@+=feaafiart1ev1aaatCvAUfeBSjuyZL2yd9gzLbvyNv2CaerbwvMCKfMBHbqedmvETj2BSbqee0evGueE0jxyaibaieYdOi=BI8qipeYdI8qiW7rqqrFfpeea0xe9LqFf0xc9q8qqaqFn0dXdHiVcFbIOFHK8Feei0lXdar=Jb9qqFfeaYRXxe9vr0=vr0=LqpWqaaeaabiGaaiaacaqabeaabeqacmaaaOqaamrtHrhAL1wy0L2yHvtyaeHbnfgDOvwBHrxAJfwnaGabaiab=ntinnaaBaaaleaacqWFge=rcqWFce=qaeqaaaaa@3BB7@	ℳGC+GU MathType@MTEF@5@5@+=feaafiart1ev1aaatCvAUfKttLearuWrP9MDH5MBPbIqV92AaeXatLxBI9gBaebbnrfifHhDYfgasaacPC6xNi=xH8viVGI8Gi=hEeeu0xXdbba9frFj0xb9qqpG0dXdb9aspeI8k8fiI+fsY=rqGqVepae9pg0db9vqaiVgFr0xfr=xfr=xc9adbaqaaeGacaGaaiaabeqaaeqabiWaaaGcbaWenfgDOvwBHrxAJfwnHbqeg0uy0HwzTfgDPnwy1aaceaGae83mH00aaSbaaSqaaiab=zq8hjab=jq8djabgUcaRiab=zq8hjab=rr8vbqabaaaaa@3EE4@	All	*M*_*HC*_	ℳGU MathType@MTEF@5@5@+=feaafiart1ev1aaatCvAUfKttLearuWrP9MDH5MBPbIqV92AaeXatLxBI9gBaebbnrfifHhDYfgasaacPC6xNi=xH8viVGI8Gi=hEeeu0xXdbba9frFj0xb9qqpG0dXdb9aspeI8k8fiI+fsY=rqGqVepae9pg0db9vqaiVgFr0xfr=xfr=xc9adbaqaaeGacaGaaiaabeqaaeqabiWaaaGcbaWenfgDOvwBHrxAJfwnHbqeg0uy0HwzTfgDPnwy1aaceaGae83mH00aaSbaaSqaaiab=zq8hjab=rr8vbqabaaaaa@3A84@	ℳGC MathType@MTEF@5@5@+=feaafiart1ev1aaatCvAUfeBSjuyZL2yd9gzLbvyNv2CaerbwvMCKfMBHbqedmvETj2BSbqee0evGueE0jxyaibaieYdOi=BI8qipeYdI8qiW7rqqrFfpeea0xe9LqFf0xc9q8qqaqFn0dXdHiVcFbIOFHK8Feei0lXdar=Jb9qqFfeaYRXxe9vr0=vr0=LqpWqaaeaabiGaaiaacaqabeaabeqacmaaaOqaamrtHrhAL1wy0L2yHvtyaeHbnfgDOvwBHrxAJfwnaGabaiab=ntinnaaBaaaleaacqWFge=rcqWFce=qaeqaaaaa@3BB7@	ℳGC+GU MathType@MTEF@5@5@+=feaafiart1ev1aaatCvAUfKttLearuWrP9MDH5MBPbIqV92AaeXatLxBI9gBaebbnrfifHhDYfgasaacPC6xNi=xH8viVGI8Gi=hEeeu0xXdbba9frFj0xb9qqpG0dXdb9aspeI8k8fiI+fsY=rqGqVepae9pg0db9vqaiVgFr0xfr=xfr=xc9adbaqaaeGacaGaaiaabeqaaeqabiWaaaGcbaWenfgDOvwBHrxAJfwnHbqeg0uy0HwzTfgDPnwy1aaceaGae83mH00aaSbaaSqaaiab=zq8hjab=jq8djabgUcaRiab=zq8hjab=rr8vbqabaaaaa@3EE4@

Avg MCC	45.19	56.82	63.38	67.66	67.45	41.03	60.27	73.58	70.13	75.3
Max MCC	89	85	84	80	85	86	84	85	80	85
Min MCC	10	26	41	56	41	5	30	56	56	56
% MCC > 75	1.3%	8.6%	5.7%	27.6%	26.7%	0.7%	18%	48.6%	23.3%	60.4%

The *SSCA *algorithm therefore gave about one chance in four of obtaining a good prediction (prediction with a MCC greater than 75) of the secondary structure of this RNA (using ℳGC
 MathType@MTEF@5@5@+=feaafiart1ev1aaatCvAUfeBSjuyZL2yd9gzLbvyNv2CaerbwvMCKfMBHbqedmvETj2BSbqee0evGueE0jxyaibaieYdOi=BI8qipeYdI8qiW7rqqrFfpeea0xe9LqFf0xc9q8qqaqFn0dXdHiVcFbIOFHK8Feei0lXdar=Jb9qqFfeaYRXxe9vr0=vr0=LqpWqaaeaabiGaaiaacaqabeaabeqacmaaaOqaamrtHrhAL1wy0L2yHvtyaeHbnfgDOvwBHrxAJfwnaGabaiab=ntinnaaBaaaleaacqWFge=rcqWFce=qaeqaaaaa@3BB7@ and ℳGC+GU
 MathType@MTEF@5@5@+=feaafiart1ev1aaatCvAUfKttLearuWrP9MDH5MBPbIqV92AaeXatLxBI9gBaebbnrfifHhDYfgasaacPC6xNi=xH8viVGI8Gi=hEeeu0xXdbba9frFj0xb9qqpG0dXdb9aspeI8k8fiI+fsY=rqGqVepae9pg0db9vqaiVgFr0xfr=xfr=xc9adbaqaaeGacaGaaiaabeqaaeqabiWaaaGcbaWenfgDOvwBHrxAJfwnHbqeg0uy0HwzTfgDPnwy1aaceaGae83mH00aaSbaaSqaaiab=zq8hjab=jq8djabgUcaRiab=zq8hjab=rr8vbqabaaaaa@3EE4@ models) while there is one chance in a hundred of success when no method of homologous sequence selection is used.

##### Results for Escherichia coli RNaseP

An alignment of 54 sequences and a reference structure provided by the RNaseP Database [25] were used.

The results were better than those for tmRNA. The models ℳGU
 MathType@MTEF@5@5@+=feaafiart1ev1aaatCvAUfKttLearuWrP9MDH5MBPbIqV92AaeXatLxBI9gBaebbnrfifHhDYfgasaacPC6xNi=xH8viVGI8Gi=hEeeu0xXdbba9frFj0xb9qqpG0dXdb9aspeI8k8fiI+fsY=rqGqVepae9pg0db9vqaiVgFr0xfr=xfr=xc9adbaqaaeGacaGaaiaabeqaaeqabiWaaaGcbaWenfgDOvwBHrxAJfwnHbqeg0uy0HwzTfgDPnwy1aaceaGae83mH00aaSbaaSqaaiab=zq8hjab=rr8vbqabaaaaa@3A84@, ℳGC
 MathType@MTEF@5@5@+=feaafiart1ev1aaatCvAUfeBSjuyZL2yd9gzLbvyNv2CaerbwvMCKfMBHbqedmvETj2BSbqee0evGueE0jxyaibaieYdOi=BI8qipeYdI8qiW7rqqrFfpeea0xe9LqFf0xc9q8qqaqFn0dXdHiVcFbIOFHK8Feei0lXdar=Jb9qqFfeaYRXxe9vr0=vr0=LqpWqaaeaabiGaaiaacaqabeaabeqacmaaaOqaamrtHrhAL1wy0L2yHvtyaeHbnfgDOvwBHrxAJfwnaGabaiab=ntinnaaBaaaleaacqWFge=rcqWFce=qaeqaaaaa@3BB7@ and ℳGC+GU
 MathType@MTEF@5@5@+=feaafiart1ev1aaatCvAUfKttLearuWrP9MDH5MBPbIqV92AaeXatLxBI9gBaebbnrfifHhDYfgasaacPC6xNi=xH8viVGI8Gi=hEeeu0xXdbba9frFj0xb9qqpG0dXdb9aspeI8k8fiI+fsY=rqGqVepae9pg0db9vqaiVgFr0xfr=xfr=xc9adbaqaaeGacaGaaiaabeqaaeqabiWaaaGcbaWenfgDOvwBHrxAJfwnHbqeg0uy0HwzTfgDPnwy1aaceaGae83mH00aaSbaaSqaaiab=zq8hjab=jq8djabgUcaRiab=zq8hjab=rr8vbqabaaaaa@3EE4@ improved the MCC average by 30% (Table 4): the average MCC is equal to 41.03 when no method for selecting homologous sequences is used and is equal to an average of 73 when using *SSCA*. The best results were obtained with the ℳGC+GU
 MathType@MTEF@5@5@+=feaafiart1ev1aaatCvAUfKttLearuWrP9MDH5MBPbIqV92AaeXatLxBI9gBaebbnrfifHhDYfgasaacPC6xNi=xH8viVGI8Gi=hEeeu0xXdbba9frFj0xb9qqpG0dXdb9aspeI8k8fiI+fsY=rqGqVepae9pg0db9vqaiVgFr0xfr=xfr=xc9adbaqaaeGacaGaaiaabeqaaeqabiWaaaGcbaWenfgDOvwBHrxAJfwnHbqeg0uy0HwzTfgDPnwy1aaceaGae83mH00aaSbaaSqaaiab=zq8hjab=jq8djabgUcaRiab=zq8hjab=rr8vbqabaaaaa@3EE4@ model. Without any method of homologous sequences selection, there was 0.7% chance of obtaining good predictions, against 60.4% when using the ℳGC+GU
 MathType@MTEF@5@5@+=feaafiart1ev1aaatCvAUfKttLearuWrP9MDH5MBPbIqV92AaeXatLxBI9gBaebbnrfifHhDYfgasaacPC6xNi=xH8viVGI8Gi=hEeeu0xXdbba9frFj0xb9qqpG0dXdb9aspeI8k8fiI+fsY=rqGqVepae9pg0db9vqaiVgFr0xfr=xfr=xc9adbaqaaeGacaGaaiaabeqaaeqabiWaaaGcbaWenfgDOvwBHrxAJfwnHbqeg0uy0HwzTfgDPnwy1aaceaGae83mH00aaSbaaSqaaiab=zq8hjab=jq8djabgUcaRiab=zq8hjab=rr8vbqabaaaaa@3EE4@ model. The ℳGC+GU
 MathType@MTEF@5@5@+=feaafiart1ev1aaatCvAUfKttLearuWrP9MDH5MBPbIqV92AaeXatLxBI9gBaebbnrfifHhDYfgasaacPC6xNi=xH8viVGI8Gi=hEeeu0xXdbba9frFj0xb9qqpG0dXdb9aspeI8k8fiI+fsY=rqGqVepae9pg0db9vqaiVgFr0xfr=xfr=xc9adbaqaaeGacaGaaiaabeqaaeqabiWaaaGcbaWenfgDOvwBHrxAJfwnHbqeg0uy0HwzTfgDPnwy1aaceaGae83mH00aaSbaaSqaaiab=zq8hjab=jq8djabgUcaRiab=zq8hjab=rr8vbqabaaaaa@3EE4@ model gave three times as many good predictions as the *M*_*HC *_model.

##### Results on other RNAs

*SSCA *was also tested for predicting the secondary structure of *Halobacterium halobium *SRP RNA, *E*chinococcus multilocularis u1RNA and *Escherichia coli *5S RNA (using the *P-DCfold *algorithm). While the tmRNA and RNaseP have around 370 nucleotides, SRP RNA, U1 RNA and 5S RNA have 300, 160 and 80 nucleotides respectively. We used a sequence alignment provided by the Signal Recognition Particle Database [32] with 54 sequences for SRP RNA, a sequence alignment from the uRNA Database [33] with 76 sequences for u1RNA and a sequence alignment from the 5S ribosomal RNA database [34] with 57 sequences for 5S RNA. The three models ℳGU
 MathType@MTEF@5@5@+=feaafiart1ev1aaatCvAUfKttLearuWrP9MDH5MBPbIqV92AaeXatLxBI9gBaebbnrfifHhDYfgasaacPC6xNi=xH8viVGI8Gi=hEeeu0xXdbba9frFj0xb9qqpG0dXdb9aspeI8k8fiI+fsY=rqGqVepae9pg0db9vqaiVgFr0xfr=xfr=xc9adbaqaaeGacaGaaiaabeqaaeqabiWaaaGcbaWenfgDOvwBHrxAJfwnHbqeg0uy0HwzTfgDPnwy1aaceaGae83mH00aaSbaaSqaaiab=zq8hjab=rr8vbqabaaaaa@3A84@, ℳGC
 MathType@MTEF@5@5@+=feaafiart1ev1aaatCvAUfeBSjuyZL2yd9gzLbvyNv2CaerbwvMCKfMBHbqedmvETj2BSbqee0evGueE0jxyaibaieYdOi=BI8qipeYdI8qiW7rqqrFfpeea0xe9LqFf0xc9q8qqaqFn0dXdHiVcFbIOFHK8Feei0lXdar=Jb9qqFfeaYRXxe9vr0=vr0=LqpWqaaeaabiGaaiaacaqabeaabeqacmaaaOqaamrtHrhAL1wy0L2yHvtyaeHbnfgDOvwBHrxAJfwnaGabaiab=ntinnaaBaaaleaacqWFge=rcqWFce=qaeqaaaaa@3BB7@ and ℳGC+GU
 MathType@MTEF@5@5@+=feaafiart1ev1aaatCvAUfKttLearuWrP9MDH5MBPbIqV92AaeXatLxBI9gBaebbnrfifHhDYfgasaacPC6xNi=xH8viVGI8Gi=hEeeu0xXdbba9frFj0xb9qqpG0dXdb9aspeI8k8fiI+fsY=rqGqVepae9pg0db9vqaiVgFr0xfr=xfr=xc9adbaqaaeGacaGaaiaabeqaaeqabiWaaaGcbaWenfgDOvwBHrxAJfwnHbqeg0uy0HwzTfgDPnwy1aaceaGae83mH00aaSbaaSqaaiab=zq8hjab=jq8djabgUcaRiab=zq8hjab=rr8vbqabaaaaa@3EE4@ provided better average MCC values than *M*_*HC *_(Table S1 in Additional file 1). About 78% of the predictions done using *SSCA *had a MCC > 75 (82% for ℳGU
 MathType@MTEF@5@5@+=feaafiart1ev1aaatCvAUfKttLearuWrP9MDH5MBPbIqV92AaeXatLxBI9gBaebbnrfifHhDYfgasaacPC6xNi=xH8viVGI8Gi=hEeeu0xXdbba9frFj0xb9qqpG0dXdb9aspeI8k8fiI+fsY=rqGqVepae9pg0db9vqaiVgFr0xfr=xfr=xc9adbaqaaeGacaGaaiaabeqaaeqabiWaaaGcbaWenfgDOvwBHrxAJfwnHbqeg0uy0HwzTfgDPnwy1aaceaGae83mH00aaSbaaSqaaiab=zq8hjab=rr8vbqabaaaaa@3A84@, 64% for ℳGC
 MathType@MTEF@5@5@+=feaafiart1ev1aaatCvAUfeBSjuyZL2yd9gzLbvyNv2CaerbwvMCKfMBHbqedmvETj2BSbqee0evGueE0jxyaibaieYdOi=BI8qipeYdI8qiW7rqqrFfpeea0xe9LqFf0xc9q8qqaqFn0dXdHiVcFbIOFHK8Feei0lXdar=Jb9qqFfeaYRXxe9vr0=vr0=LqpWqaaeaabiGaaiaacaqabeaabeqacmaaaOqaamrtHrhAL1wy0L2yHvtyaeHbnfgDOvwBHrxAJfwnaGabaiab=ntinnaaBaaaleaacqWFge=rcqWFce=qaeqaaaaa@3BB7@ and 88% for ℳGC+GU
 MathType@MTEF@5@5@+=feaafiart1ev1aaatCvAUfKttLearuWrP9MDH5MBPbIqV92AaeXatLxBI9gBaebbnrfifHhDYfgasaacPC6xNi=xH8viVGI8Gi=hEeeu0xXdbba9frFj0xb9qqpG0dXdb9aspeI8k8fiI+fsY=rqGqVepae9pg0db9vqaiVgFr0xfr=xfr=xc9adbaqaaeGacaGaaiaabeqaaeqabiWaaaGcbaWenfgDOvwBHrxAJfwnHbqeg0uy0HwzTfgDPnwy1aaceaGae83mH00aaSbaaSqaaiab=zq8hjab=jq8djabgUcaRiab=zq8hjab=rr8vbqabaaaaa@3EE4@), when only 46% of the predictions done using the method *M*_*HC *_had a MCC > 75. This means that there was about three chances in four of obtaining a good prediction when using *SSCA*. The best results were, again, obtained with ℳGC+GU
 MathType@MTEF@5@5@+=feaafiart1ev1aaatCvAUfKttLearuWrP9MDH5MBPbIqV92AaeXatLxBI9gBaebbnrfifHhDYfgasaacPC6xNi=xH8viVGI8Gi=hEeeu0xXdbba9frFj0xb9qqpG0dXdb9aspeI8k8fiI+fsY=rqGqVepae9pg0db9vqaiVgFr0xfr=xfr=xc9adbaqaaeGacaGaaiaabeqaaeqabiWaaaGcbaWenfgDOvwBHrxAJfwnHbqeg0uy0HwzTfgDPnwy1aaceaGae83mH00aaSbaaSqaaiab=zq8hjab=jq8djabgUcaRiab=zq8hjab=rr8vbqabaaaaa@3EE4@ model: about 90% of the predictions were good when using this model for selecting homologous sequences.

#### Results obtained using the RNAalifold algorithm

We tested whether *SSCA *was suitable for use with another RNA secondary structure prediction algorithm, the *RNAalifold *[16] algorithm. Since *RNAalifold *computes a consensus structure and since we wanted to obtain a structure for one sequence, we obtained the best thermodynamic folding for this sequence that had the base-pairs specified by the constraint of the consensus structure. This refolding was done by RNAfold with option -C.

We tested *RNAalifold *on tmRNA and RNaseP alignments, using the same procedure than the one used for *P-DCfold*. We have considered three cases: all sequences of the alignment, ten sequences selected by the model *M*_*HC *_and ten sequences selected by the model ℳGC+GU
 MathType@MTEF@5@5@+=feaafiart1ev1aaatCvAUfKttLearuWrP9MDH5MBPbIqV92AaeXatLxBI9gBaebbnrfifHhDYfgasaacPC6xNi=xH8viVGI8Gi=hEeeu0xXdbba9frFj0xb9qqpG0dXdb9aspeI8k8fiI+fsY=rqGqVepae9pg0db9vqaiVgFr0xfr=xfr=xc9adbaqaaeGacaGaaiaabeqaaeqabiWaaaGcbaWenfgDOvwBHrxAJfwnHbqeg0uy0HwzTfgDPnwy1aaceaGae83mH00aaSbaaSqaaiab=zq8hjab=jq8djabgUcaRiab=zq8hjab=rr8vbqabaaaaa@3EE4@ of *SSCA*. Then we performed predictions with *RNAalifold *for each combination of 4 sequences. The results obtained are given in Table 5. The ℳGC+GU
 MathType@MTEF@5@5@+=feaafiart1ev1aaatCvAUfKttLearuWrP9MDH5MBPbIqV92AaeXatLxBI9gBaebbnrfifHhDYfgasaacPC6xNi=xH8viVGI8Gi=hEeeu0xXdbba9frFj0xb9qqpG0dXdb9aspeI8k8fiI+fsY=rqGqVepae9pg0db9vqaiVgFr0xfr=xfr=xc9adbaqaaeGacaGaaiaabeqaaeqabiWaaaGcbaWenfgDOvwBHrxAJfwnHbqeg0uy0HwzTfgDPnwy1aaceaGae83mH00aaSbaaSqaaiab=zq8hjab=jq8djabgUcaRiab=zq8hjab=rr8vbqabaaaaa@3EE4@ model gave average MCC values higher than the ones obtained by the *M*_*HC *_model which in turn are better than the ones obtained when no method for homologous sequences selection is used.

**Table 5:** Average MCC distributions of tmRNA and RNaseP secondary structure predictions done with the *RNAalifold *algorithm and using the model *M*_*HC *_and the model ℳGC+GU
 MathType@MTEF@5@5@+=feaafiart1ev1aaatCvAUfKttLearuWrP9MDH5MBPbIqV92AaeXatLxBI9gBaebbnrfifHhDYfgasaacPC6xNi=xH8viVGI8Gi=hEeeu0xXdbba9frFj0xb9qqpG0dXdb9aspeI8k8fiI+fsY=rqGqVepae9pg0db9vqaiVgFr0xfr=xfr=xc9adbaqaaeGacaGaaiaabeqaaeqabiWaaaGcbaWenfgDOvwBHrxAJfwnHbqeg0uy0HwzTfgDPnwy1aaceaGae83mH00aaSbaaSqaaiab=zq8hjab=jq8djabgUcaRiab=zq8hjab=rr8vbqabaaaaa@3EE4@ of *SSCA *for selecting homologous sequences.

	All sequences	*M*_*HC*_	ℳGC+GU MathType@MTEF@5@5@+=feaafiart1ev1aaatCvAUfKttLearuWrP9MDH5MBPbIqV92AaeXatLxBI9gBaebbnrfifHhDYfgasaacPC6xNi=xH8viVGI8Gi=hEeeu0xXdbba9frFj0xb9qqpG0dXdb9aspeI8k8fiI+fsY=rqGqVepae9pg0db9vqaiVgFr0xfr=xfr=xc9adbaqaaeGacaGaaiaabeqaaeqabiWaaaGcbaWenfgDOvwBHrxAJfwnHbqeg0uy0HwzTfgDPnwy1aaceaGae83mH00aaSbaaSqaaiab=zq8hjab=jq8djabgUcaRiab=zq8hjab=rr8vbqabaaaaa@3EE4@
tmRNA	52,54	58,17	60,09
RNaseP	58,92	60,93	65,37

#### SSCA and MCC scores

We checked the capacity of the ℳGC+GU
 MathType@MTEF@5@5@+=feaafiart1ev1aaatCvAUfKttLearuWrP9MDH5MBPbIqV92AaeXatLxBI9gBaebbnrfifHhDYfgasaacPC6xNi=xH8viVGI8Gi=hEeeu0xXdbba9frFj0xb9qqpG0dXdb9aspeI8k8fiI+fsY=rqGqVepae9pg0db9vqaiVgFr0xfr=xfr=xc9adbaqaaeGacaGaaiaabeqaaeqabiWaaaGcbaWenfgDOvwBHrxAJfwnHbqeg0uy0HwzTfgDPnwy1aaceaGae83mH00aaSbaaSqaaiab=zq8hjab=jq8djabgUcaRiab=zq8hjab=rr8vbqabaaaaa@3EE4@ model to measure the usefulness of sequences for the comparative approach. We used the two alignments of tmRNA and RNaseP to predict the secondary structures of *Escherichia Coli *sequences for all possible combinations of four homologous sequences among all the homologous sequences of the alignments, using the algorithm *P-DCfold*. We calculated the average MCC score for each homologous sequence. The most useful sequences for predicting the secondary structure had the highest average MCC scores.

**Figure 4:**
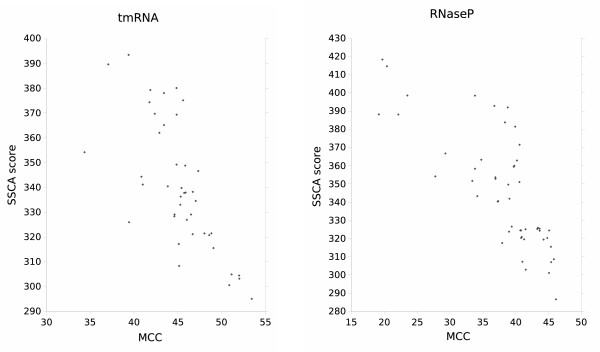
Correlation between *SSCA *scores (using the model ℳGC+GU
 MathType@MTEF@5@5@+=feaafiart1ev1aaatCvAUfKttLearuWrP9MDH5MBPbIqV92AaeXatLxBI9gBaebbnrfifHhDYfgasaacPC6xNi=xH8viVGI8Gi=hEeeu0xXdbba9frFj0xb9qqpG0dXdb9aspeI8k8fiI+fsY=rqGqVepae9pg0db9vqaiVgFr0xfr=xfr=xc9adbaqaaeGacaGaaiaabeqaaeqabiWaaaGcbaWenfgDOvwBHrxAJfwnHbqeg0uy0HwzTfgDPnwy1aaceaGae83mH00aaSbaaSqaaiab=zq8hjab=jq8djabgUcaRiab=zq8hjab=rr8vbqabaaaaa@3EE4@) and average MCC scores of homologous sequences of tmRNA (left) and RNaseP (right) alignments. Homologous sequences with the lowest *SSCA *scores have the highest average MCC scores. The best correlation is for the low *SSCA *scores.

We plotted the correlation between the average MCC score of each homologous sequence and the score *S*_*GC*+*GU *_attributed to this sequence with the model ℳGC+GU
 MathType@MTEF@5@5@+=feaafiart1ev1aaatCvAUfKttLearuWrP9MDH5MBPbIqV92AaeXatLxBI9gBaebbnrfifHhDYfgasaacPC6xNi=xH8viVGI8Gi=hEeeu0xXdbba9frFj0xb9qqpG0dXdb9aspeI8k8fiI+fsY=rqGqVepae9pg0db9vqaiVgFr0xfr=xfr=xc9adbaqaaeGacaGaaiaabeqaaeqabiWaaaGcbaWenfgDOvwBHrxAJfwnHbqeg0uy0HwzTfgDPnwy1aaceaGae83mH00aaSbaaSqaaiab=zq8hjab=jq8djabgUcaRiab=zq8hjab=rr8vbqabaaaaa@3EE4@ (Figure 4). Figure 4 shows that the homologous sequences with the lowest *SSCA *scores have the highest average MCC scores, validating our model and algorithm for selecting homologous sequences.

We did a correlation study using Pearson's correlation coefficient, which is a measure of correlation between two variables. This coefficient varies between -1 for variables inversely correlated (which is the case here) and 1 for variables correlated in the same way. When it is equal to 0, this means that the two variables are not correlated. On our data, we obtained 0.68 for tmRNA and 0.79 for RNaseP, showing the inverse correlation existing between *SSCA *scores and MCC.

## Discussion and Conclusion

We have developed an algorithm, *SSCA*, for selecting homologous sequences for use in predicting the secondary structure of RNAs using the comparative approach. Homologous sequences selection is based on the idea that structure constraints skew the substitution matrices in stems. We have defined three selection models, ℳGU
 MathType@MTEF@5@5@+=feaafiart1ev1aaatCvAUfKttLearuWrP9MDH5MBPbIqV92AaeXatLxBI9gBaebbnrfifHhDYfgasaacPC6xNi=xH8viVGI8Gi=hEeeu0xXdbba9frFj0xb9qqpG0dXdb9aspeI8k8fiI+fsY=rqGqVepae9pg0db9vqaiVgFr0xfr=xfr=xc9adbaqaaeGacaGaaiaabeqaaeqabiWaaaGcbaWenfgDOvwBHrxAJfwnHbqeg0uy0HwzTfgDPnwy1aaceaGae83mH00aaSbaaSqaaiab=zq8hjab=rr8vbqabaaaaa@3A84@, based on GU intermediate state constraints, ℳGC
 MathType@MTEF@5@5@+=feaafiart1ev1aaatCvAUfeBSjuyZL2yd9gzLbvyNv2CaerbwvMCKfMBHbqedmvETj2BSbqee0evGueE0jxyaibaieYdOi=BI8qipeYdI8qiW7rqqrFfpeea0xe9LqFf0xc9q8qqaqFn0dXdHiVcFbIOFHK8Feei0lXdar=Jb9qqFfeaYRXxe9vr0=vr0=LqpWqaaeaabiGaaiaacaqabeaabeqacmaaaOqaamrtHrhAL1wy0L2yHvtyaeHbnfgDOvwBHrxAJfwnaGabaiab=ntinnaaBaaaleaacqWFge=rcqWFce=qaeqaaaaa@3BB7@, based on GC stability constraints and ℳGC+GU
 MathType@MTEF@5@5@+=feaafiart1ev1aaatCvAUfKttLearuWrP9MDH5MBPbIqV92AaeXatLxBI9gBaebbnrfifHhDYfgasaacPC6xNi=xH8viVGI8Gi=hEeeu0xXdbba9frFj0xb9qqpG0dXdb9aspeI8k8fiI+fsY=rqGqVepae9pg0db9vqaiVgFr0xfr=xfr=xc9adbaqaaeGacaGaaiaabeqaaeqabiWaaaGcbaWenfgDOvwBHrxAJfwnHbqeg0uy0HwzTfgDPnwy1aaceaGae83mH00aaSbaaSqaaiab=zq8hjab=jq8djabgUcaRiab=zq8hjab=rr8vbqabaaaaa@3EE4@, based on both GC stability and GU intermediate state constraints.

We compared our three models with a currently used model (*M*_*HC*_) by predicting the secondary structures of tmRNA and RNaseP using *P-DCfold *algorithm. All the three models significantly improved the probability of obtaining good predictions. The three models, ℳGU
 MathType@MTEF@5@5@+=feaafiart1ev1aaatCvAUfKttLearuWrP9MDH5MBPbIqV92AaeXatLxBI9gBaebbnrfifHhDYfgasaacPC6xNi=xH8viVGI8Gi=hEeeu0xXdbba9frFj0xb9qqpG0dXdb9aspeI8k8fiI+fsY=rqGqVepae9pg0db9vqaiVgFr0xfr=xfr=xc9adbaqaaeGacaGaaiaabeqaaeqabiWaaaGcbaWenfgDOvwBHrxAJfwnHbqeg0uy0HwzTfgDPnwy1aaceaGae83mH00aaSbaaSqaaiab=zq8hjab=rr8vbqabaaaaa@3A84@, ℳGC
 MathType@MTEF@5@5@+=feaafiart1ev1aaatCvAUfeBSjuyZL2yd9gzLbvyNv2CaerbwvMCKfMBHbqedmvETj2BSbqee0evGueE0jxyaibaieYdOi=BI8qipeYdI8qiW7rqqrFfpeea0xe9LqFf0xc9q8qqaqFn0dXdHiVcFbIOFHK8Feei0lXdar=Jb9qqFfeaYRXxe9vr0=vr0=LqpWqaaeaabiGaaiaacaqabeaabeqacmaaaOqaamrtHrhAL1wy0L2yHvtyaeHbnfgDOvwBHrxAJfwnaGabaiab=ntinnaaBaaaleaacqWFge=rcqWFce=qaeqaaaaa@3BB7@ and ℳGC+GU
 MathType@MTEF@5@5@+=feaafiart1ev1aaatCvAUfKttLearuWrP9MDH5MBPbIqV92AaeXatLxBI9gBaebbnrfifHhDYfgasaacPC6xNi=xH8viVGI8Gi=hEeeu0xXdbba9frFj0xb9qqpG0dXdb9aspeI8k8fiI+fsY=rqGqVepae9pg0db9vqaiVgFr0xfr=xfr=xc9adbaqaaeGacaGaaiaabeqaaeqabiWaaaGcbaWenfgDOvwBHrxAJfwnHbqeg0uy0HwzTfgDPnwy1aaceaGae83mH00aaSbaaSqaaiab=zq8hjab=jq8djabgUcaRiab=zq8hjab=rr8vbqabaaaaa@3EE4@ gave better results than the model *M*_*HC*_. The better model is ℳGC+GU
 MathType@MTEF@5@5@+=feaafiart1ev1aaatCvAUfKttLearuWrP9MDH5MBPbIqV92AaeXatLxBI9gBaebbnrfifHhDYfgasaacPC6xNi=xH8viVGI8Gi=hEeeu0xXdbba9frFj0xb9qqpG0dXdb9aspeI8k8fiI+fsY=rqGqVepae9pg0db9vqaiVgFr0xfr=xfr=xc9adbaqaaeGacaGaaiaabeqaaeqabiWaaaGcbaWenfgDOvwBHrxAJfwnHbqeg0uy0HwzTfgDPnwy1aaceaGae83mH00aaSbaaSqaaiab=zq8hjab=jq8djabgUcaRiab=zq8hjab=rr8vbqabaaaaa@3EE4@, which gave three times more good predictions than the model *M*_*HC *_(Tables 3).

We also tested *SSCA *on other RNAs: RP RNA, U1 RNA and 5S RNA, and we obtained good results. Finally, we tested the use of *SSCA *with *RNAalifold *algorithm. The results when *SSCA *was used for selecting homologous sequences were also better than when no selection of homologous sequences is done. Nevertheless, the results obtained with *RNAalifold *were not as good as the ones obtained with *P-DCfold*. One reason could be because *RNAalifold *was designed and optimized for predicting a common secondary structure of a set of homologous sequences, instead of *P-DCfold *which was designed and optimized for predicting the secondary structure of one sequence using a set of homologous sequences.

To improve the results of predictions, it may be possible to make several predictions using different subsets of homologous sequences selected by *SSCA *and to calculate the structure common to these predictions. Our basic idea of selecting sequences with adequate variability can be improved by using phylogenetic trees. Skimming through the phylogenetic tree can select homologous sequences that are variable and similar enough to the target sequence.

Finally, the time complexity of the *SSCA *algorithm is O
 MathType@MTEF@5@5@+=feaafiart1ev1aaatCvAUfKttLearuWrP9MDH5MBPbIqV92AaeXatLxBI9gBaebbnrfifHhDYfgasaacPC6xNi=xH8viVGI8Gi=hEeeu0xXdbba9frFj0xb9qqpG0dXdb9aspeI8k8fiI+fsY=rqGqVepae9pg0db9vqaiVgFr0xfr=xfr=xc9adbaqaaeGacaGaaiaabeqaaeqabiWaaaGcbaWenfgDOvwBHrxAJfwnHbqeg0uy0HwzTfgDPnwy1aaceaqcfaOae8NdX=eaaa@37F1@(*m *× *n*), with *n *the length of the target sequence and *m *the number of homologous sequences. All our tests took less than 5 seconds.

## Authors' contributions

The work presented in this paper is part of the PhD Thesis of SE, directed by FT. SE carried out the tests and analyzed the results under the direction of FT. SE and FT drafted the manuscript. They have both read and approved the final manuscript.

## Supplementary Material

Additional file 1 Average MCC distributions of secondary structure predictions done with the *P-DCfold *algorithm and using different homologous sequence selection models on SRP RNA, U1 RNA and 5S RNA. Click here for file
