# Supplementary material for: Predicting RNA secondary structure by the comparative approach: how to select the homologous sequences
Source: BMC Bioinformatics. 2007 Nov 28;8:464. doi: 10.1186/1471-2105-8-464 (PMC2238770; doi:10.1186/1471-2105-8-464)
Supplement: Additional file 1 — Average MCC distributions of secondary structure predictions done with the P-DCfold algorithm and using different homologous sequence selection models on SRP RNA, U1 RNA and 5S RNA. [file 1471-2105-8-464-S1.pdf]

Table S1 - Average MCC distributions of secondary structure predictions done with the *P-DCfold* algorithm and using different homologous sequence selection models on SRP RNA (top), U1 RNA (center) and 5S RNA (bottom).

	All	M_{HC}	$\mathcal{M}_{\mathcal{GU}}$	$\mathcal{M}_{\mathcal{GC}}$	$\mathcal{M}_{\mathcal{GC}+\mathcal{GU}}$
Avg MCC	51.83	68.98	73.74	67.23	75.39
Max MCC	91	90	89	90	85
Min MCC	3	37	40	37	45
% of predictions with MCC>75	6.4%	33%	61%	24%	67%

	All	M_{HC}	$\mathcal{M}_{\mathcal{GU}}$	$\mathcal{M}_{\mathcal{GC}}$	$\mathcal{M}_{\mathcal{GC}+\mathcal{GU}}$
Avg MCC	62.56	74.02	84.05	80.95	84.63
Max MCC	96	93	93	93	93
Min MCC	4	28	49	55	57
% of predictions with MCC>75	25.7%	61%	95%	70%	96%

	All	M_{HC}	$\mathcal{M}_{\mathcal{GU}}$	$\mathcal{M}_{\mathcal{GC}}$	$\mathcal{M}_{\mathcal{GC}+\mathcal{GU}}$
Avg MCC	79.48	70.58	84.78	90.1	90.12
Max MCC	97	92	95	94	92
Min MCC	14	19	74	74	81
% of predictions with MCC>75	67.7%	45%	90%	98%	100%
